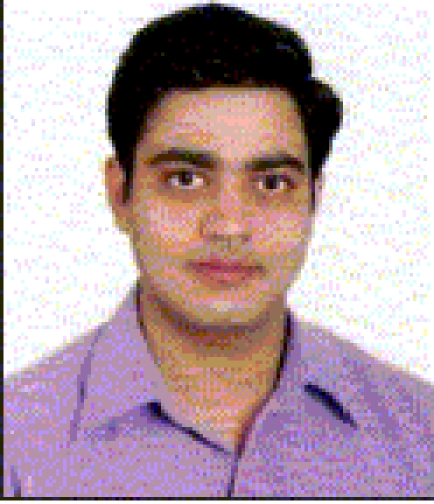# Launch of the special issue of JPBS on CBRN disaster management

**DOI:** 10.4103/0975-7406.76460

**Published:** 2011

**Authors:** Himanshu Gupta

**Affiliations:** Managing Editor - JPBS 21, Jaina Building, Roshanara Road, Delhi-110007, India E-mail: hgupta@jpbsonline.org


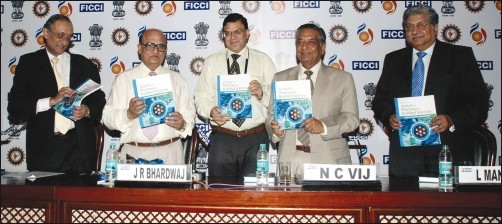


New Delhi, India, August 30, 2010 - The Journal of Pharmacy and BioAllied Sciences (JPBS) today announced the launch of a special issue on Chemical, Biological, Radiological, and Nuclear (CBRN) Disaster Management,, edited by Dr. Rakesh Kumar Sharma, Scientist ‘G’, Additional Director and Head, CBRN Defence, Institute of Nuclear Medicine and Allied Sciences (INMAS), Defence Research and Development Organization, Brig. SK Mazumdar Marg, Delhi, India. Lt. Gen. J. R. Bhardwaj, Honorable Member, National Disaster Management Authority released the issue on August 30, 2010, during the inauguration of the Conference on ’Emergency Planning in Industries including Halma Water Management (HWM) and Transportation of Petroleum, Petroleum Products, Natural Gas by Pipelines and Petroleum, Oil, and Lubricant (POL) Tankers,’ organized by the Federation of Indian Chambers of Commerce and Industry (FICCI), in association with the National Disaster Management Authority (NDMA) and Ministry of Environment and Forests (MoEF), Government of India, and the Petroleum and Natural Gas Regulatory Board (PNGRB).

Release of the Special Issue of JPBS on CBRN Disaster Management. Seen in the picture from left to right are Dr. Amit Mitra, Secretary General, FICCI, Lt. Gen. J. R. Bhardwaj, Honorable Member, NDMA, Dr. Rakesh Kumar Sharma, Additional Director - CBRN Defence, INMAS / DRDO and Editor of the Special Issue of JPBS, Gen. N C Vij, Honorable Vice Chairman, NDMA and Shri L. Mansingh, Chairperson of the Petroleum and Natural Gas Regulatory Board.

For further information please contact